# Job satisfaction among primary care physicians in western China

**DOI:** 10.1186/s12875-020-01292-w

**Published:** 2020-10-25

**Authors:** Zhuo Zhang, Guoshuai Shi, Lingui Li, Ying Bian

**Affiliations:** 1 State Key Laboratory of Quality Research in Chinese Medicine, Institute of Chinese Medical Sciences, University of Macau, Taipa, 999078 Macau China; 2 School of Health Services Management, Xi’an Medical University, Xi’an, 710021 Shaanxi China; 3 Department of Epidemiology and Biostatistics, School of Public Health, Xi’an Jiaotong University Health Science Center, Xi’an, 710061 Shaanxi China; 4 Zhejiang Chinese Medical University, Hangzhou, China

**Keywords:** Primary care physician, Job satisfaction, Quantile regression, Western China

## Abstract

**Background:**

There has been great shortage of primary care physicians (PCPs) in China, especially in western areas. Job satisfaction plays a great role in retaining people. The aim of this study is to investigate the job satisfaction of PCPs and associated factors in 11 provinces of western China, thus providing necessary reference values for stabilizing the primary care workforce and improving the quality of primary care services.

**Method:**

A sample of 2103 PCPs working in western China were surveyed using a stratified, multistage and random sampling method in 2011. The characteristics of participants were recorded by a structured questionnaire. A multilevel model (MLM) and quantile regression (QR) were applied to assess the association between job satisfaction and possible risk factors.

**Results:**

Of the 2103 doctors surveyed, the overall satisfaction score was 3.26 ± 0.68 (from 1 to 5). MLM indicated that age group, income satisfaction, unit policy approval, personal planning, career attitude, work value and patient recognition were positively correlated with job satisfaction, while turnover intention was negatively correlated with job satisfaction. QR were not completely consistent with MLM and further explored the differences in different job satisfaction score percentiles on each domains.

**Conclusion:**

This study showed that the job satisfaction of PCPs in western China was not high. The MLM and QR discussed were not entirely consistent, the latter one provided more information and robust results. Measures should be taken in streamlining administration and institute decentralization, creating more opportunities for additional training, raising PCPs’ income, improving the social status of doctors and improving the relationship between doctors and patients.

## Background

Job satisfaction is defined as an evaluation of the staff’s emotional state, whether or not they like the job [1]. Since the concept of job satisfaction was proposed by Hoppock, it has been widely studied in organizational research and it is a basic target for all the organizations to achieve at any time based on the fact that job satisfaction is related to productivity, absenteeism, turnover, and organizational citizenship [2]. Higher satisfaction of medical staff helps stabilize the medical service team, improve the two-way satisfaction of doctors and patients, and build a harmonious doctor-patient relationship, so as to ensure the quality and efficiency of medical services, and promote the implementation of medical reform policies [3].

Primary health care is the focus of China’s health care reform and the dual net of either China’s medical or public healthcare system. Xi Li’s study noted vital importance of primary care in achieving the goals of fairness and efficiency in health care [4]. It was proposed that the Chinese government’s main obstacle to improve primary health care was the human resource crisis [5]. PCPs are the main force of China’s primary care teams, special attention has been paid to the retention of PCP working in rural remote areas. PCPs in rural western China have poor working environment and scarce health resources, poor wages and benefits. Moreover, there are few opportunities for continuing education and further studies for mastering medical technology and equipment, and less career development opportunities. In this difficult situation, how to retain them is a very important issue for related health service managers and policy makers. Job satisfaction played an important role in attracting talents, improving the job recognition and service ability of PCPs. Large number of studies have demonstrated that job satisfaction plays a key role in the retention of PCPs [6–8]. Therefore, research on the influencing factors of job satisfaction among PCPs has important practical significance.

More and more studies have demonstrated the fact that PCPs’ job satisfaction is not high. Gu’s study confirmed that PCPs in rural Shandong were not satisfied with the job [9]. Ab Rahman’s investigation conducted in Malaysia indicated that only 62.9% of PCPs believed their effort and income were consistent [10]. Meanwhile, a systematic review among urban community health workers in China and a study conducted in Chongqing showed the reduction of income and other reasons such as limited clinical autonomy caused by the essential drug list adversely affected the doctors’ job satisfaction and it had been a serious brain drain from basic medical institutions [11, 12]. A large number of studies have indicated that improving job satisfaction of PCPs is an important measure to maintain the stability of primary health team and curb the brain drain that is jeopardizing western development campaign and the aim to provide “universal healthcare by 2020” which focuses on balanced allocation of healthcare resources to rural, remote, poor and ethnic minority areas [9, 12, 13].

Satisfaction survey among PCPs is essential for patients, doctors and medical insurance payers. The main difficulties in China’s reform lie in rural areas and primary health care provision [9]. So far, limited studies were designed to explore PCPs’ satisfaction with a large-sample evidence covering different backward provinces, namely northwest and southwest of China. Considering the relatively more complex cultural, natural and socioeconomic conditions where more than fifty minorities living together, and the lower education level, less individual income, and heavier economic burden in the western part of the country than in the eastern and central areas, it is necessary to conduct a region-specific questionnaire survey for the PCPs’ satisfaction in rural western China. Additionally, despite the increasing attention on the doctors’ job satisfactory, no studies carried out in China about factors affecting job satisfaction of PCPs using a quantile regression analysis. Therefore, the aim of this study is to evaluate job satisfaction among PCPs in rural area of western China based on a quantile regression analysis following the health system reforms in 2009 and examine the determinants of job satisfaction.

## Methods

### Data and participants

The data used in this study was from a large population-based cross-sectional survey conducted in 2011, in 11 provinces of northwest China including Ningxia Hui Autonomous Region, Guangxi Zhuang Autonomous Region, Xinjiang Weiwuer Autonomous Region, Gansu Province, Shaanxi province, Qinghai province, Sichuan province, Guizhou province, Yunnan province, Inner Mongolia, and Tibet Autonomous Region. In each province, all counties were divided into 3 levels by GDP per capital (high, moderate, and low GDP per capita). One county was randomly selected from each level. Thirty-three counties were eventually included in the study. Then in each sample county, health-care facilities including a general hospital, a maternal and child health center, a hospital of Traditional Chinese Medicine and a center of disease control were recruited as sample hospitals. In each sample health facilities, 50 PCPs were drawn randomly and enrolled into the questionnaire survey. And finally, a total of 2103 PCPs were selected with response rate 95.59%.

### Data collection

Prior to the study, trained students from local medical colleges introduced the background and main purpose of the survey to the enrolled PCPs. After the written informed consent was signed, the investigators used a structured questionnaire to collect information about the respondents. The participants completed the questionnaire independently. The questionnaire was anonymous and data obtained was for research use only and kept confidential.

All investigators had undergone uniform training to understand the purpose of the investigation and the investigation process, then conduct the investigation after passing the inspection. An investigation manual was prepared, and all on-site work was carried out in accordance with the investigation manual. After the questionnaire was completed, the supervisor would check it, and return to the investigators if any problems such as logic errors or missing data were found.

### Measurement

In this study, the overall job satisfaction of the doctors was considered to be the main output. The questionnaire included 13 items which was designed as a 5-point Likert scale, and interviewees were asked to rate each item: very dissatisfied (1), dissatisfied (2), neither satisfied nor dissatisfied (3), satisfied (4), and very satisfied (5). Satisfaction issues mainly include several aspects: job fulfillment, relationships with superiors, employee recognition, job security, superiors’ decision-making ability, institution policy implement, opportunity to utilize skills and talents, chance of promotion, remuneration compared to workload, working environment, utilization of subjective initiative, sense of achievement and relationship with colleagues. The reliability and validity of the questionnaire have been confirmed in homogeneous study [14]. Job satisfaction includes two dimensions: management satisfaction (Satisfaction with institutional policies and managerial decisions) and job return satisfaction (Satisfaction with work remuneration and work accomplishment) [14].

### Covariates

In this study, we evaluated information regarding doctors’ Socio-demographic characteristics including gender (male or female), age (≤26, 27 ~ 45 or ≥ 46), marital status (single, married or widowed or divorced), education level (below college, college, bachelor or master), work seniority (≤15, 16 ~ 30 or ≥ 31), professional title (junior, middle or senior), income satisfaction (dissatisfied, average or satisfied), turnover intention (no or yes), work stress (no, mild, moderate, severe or very severe), career planning and professional identity as potential risk factors for job satisfaction. Career planning and professional identity were also designed as a 5-point Likert scale. The career planning questionnaire (The process of continuous and systematic planning of career) included two dimensions of unit policy approval and personal planning, with a total of 11 items such as I set a long-term goal for my career development and I think there are a lot of training and development opportunities. The cronbach’s α value of the questionnaire was 0.702, indicating that the questionnaire has a good reliability. The professional identify (The extent to which an individual considers his professional role to be important, attractive, and harmonious with other roles) questionnaire included three dimensions of career attitude, work value and patient recognition, with a total of 13 items such as I feel that patients respect me and I have full confidence in the development of the unit. The reliability results was shown in another article [15].

### Statistical analysis

All data were manually checked for completeness and double-entered into Microsoft Excel 2013. The continuous variables were described by mean ± SD, and Student *t* test or variance analysis was used for different group comparison. The categorical variables were described using counts and proportions.

The intra-class correlation (ICC = 0.083) was statistically significant (*P* < 0.001) after running the empty models which indicated that there was homogeneity in the job satisfaction, the multilevel model (MLM) was used to analysis the relationships between job satisfaction and potential risk factors with provinces set to level 2 and individuals set to level 1 using the meglm process in Stata 15.0 software.

The traditional regression model could only get the influence of relevant factors on the job satisfaction expectation value. However, by assessing the family of conditional quantile function, the quantile regression (QR) offered a complete image of the influences of covariates on job satisfaction. Therefore, the hierarchical QR model was employed further in this study with provinces set to level 2 and individuals set to level 1, which lead to more comprehensive analysis results. The internal point method was used to fit the QR model of job satisfaction at different percentile (q = 0.10, 0.20, 0.30, 0.40, 0.50, 0.60 0.70, 0.80, 0.90).

All data analysis was performed using Stata 15.0. Statistically significant was defined with two-tailed *p* < 0.05.

## Results

### Baseline characteristics of study population

Table 1 summarized the sociodemographic characteristics. Of the 2103 PCPS surveyed, Male accounted for 49.95% and the average age was 34.74 ± 9.20 years. 78.41% were married. The proportions of junior title, middle title and senior title of the participants were 65.19, 26.53 and 8.27%, respectively. The average work seniority was 11.26 ± 9.21 years. The doctors’ education level mainly concentrated in bachelor and college (83.88%).

**Table 1:** Characteristics of the participants

	N	P (%)
Gender		
Male	1041	49.50
Female	1062	50.50
Age (year)		
≤ 26	428	20.35
27 ~ 45	1387	65.95
≥ 46	288	13.69
Marital status		
Single	402	19.12
Married	1649	78.41
Widowed or divorced	52	2.47
Professional title		
Junior	1371	65.19
Middle	558	26.53
Senior	174	8.27
work seniority (year)		
≤ 15	1479	70.33
16 ~ 30	554	26.34
≥ 31	70	3.33
Education background		
Secondary school or below	320	15.22
College	880	41.84
Bachelor	884	42.04
Master	19	0.90

### Status of job satisfaction, career planning and professional identity scores

The overall satisfaction score of 2103 PCPS was 3.26 ± 0.68, among which, the satisfaction score of organizational management and job return were 3.41 ± 0.74 and 3.09 ± 0.77, respectively. Doctors from Gansu Province had the lowest score (2.86 ± 0.68), while those from Inner Mongolia Autonomous Region had the highest score (3.64 ± 0.71). The difference was statistically significant (P<0.05). In terms of career planning, the unit policy approval score and personal planning score were 3.03 ± 0.82 and 3.21 ± 0.80, respectively. In terms of professional identity, the sore of career attitude, work value and patient recognition were 3.55 ± 0.83, 3.70 ± 0.79 and 3.75 ± 0.78, respectively (Table 2).

**Table 2:** Status of job satisfaction, career planning and professional identity score

	Mean ± SD
Overall satisfaction	3.26 ± 0.68
Management satisfaction	3.41 ± 0.74
Job return satisfaction	3.09 ± 0.77
Career planning	3.12 ± 0.68
Unit policy approval	3.03 ± 0.82
Personal planning	3.21 ± 0.80
Professional identity	3.67 ± 0.70
Career attitude	3.55 ± 0.83
Work value	3.70 ± 0.79
Patient recognition	3.75 ± 0.78

### Univariate analysis of possible influencing factors on overall satisfaction

Univariate analysis results showed that PCPs’ age, education level, income satisfaction, turnover intention and a total of 7 factors had significantly statistical effects on job satisfaction score. Job satisfaction and it two dimensions were significantly positively correlated with the dimensions of career planning and professional identity (*P* < 0.05) (Table 3).

**Table 3:** Univariate analysis of possible influencing factors on overall satisfaction

	Mean ± SD	t/F/r	***P*** values
Gender		0.58	0.561
Male	3.27 ± 0.71		
Female	3.25 ± 0.64		
Age (year) ^1^		12.46	0.000
≤26	3.15 ± 0.68		
27 ~ 45	3.27 ± 0.68		
≥46	3.40 ± 0.68		
Marital status		2.59	0.075
Single	3.19 ± 0.72		
Married	3.28 ± 0.67		
Widowed or divorced	3.27 ± 0.76		
Education level ^1^		3.46	0.016
Below college	3.33 ± 0.63		
College	3.29 ± 0.68		
Bachelor	3.22 ± 0.69		
Master	3.08 ± 0.92		
Work seniority (year) ^1^		6.80	0.001
≤15	3.23 ± 0.69		
16 ~ 30	3.33 ± 0.66		
≥31	3.45 ± 0.52		
Professional title ^1^		5.57	0.004
Junior	3.24 ± 0.68		
Middle	3.28 ± 0.68		
Senior	3.42 ± 0.69		
Income satisfaction ^1^		63.68	0.000
Dissatisfied	3.13 ± 0.66		
Average	3.32 ± 0.61		
Satisfied	3.63 ± 0.79		
Turnover intention^1^		14.52	0.000
No	3.41 ± 0.66		
Yes	2.97 ± 0.62		
Work stress ^1^		4.21	0.000
No	3.37 ± 0.82		
Mild	3.32 ± 0.67		
Moderate	3.30 ± 0.68		
Severe	3.26 ± 0.64		
Very severe	3.15 ± 0.70		
Career planning			
Unit policy approval ^1^		0.64	0.000
Personal planning ^1^		0.41	0.000
Professional identity			
Career attitude ^1^		0.57	0.000
Work value ^1^		0.64	0.000
Patient recognition ^1^		0.49	0.000

Note:^1^ *P* < 0.05

### Multivariate analysis of the determinants of job satisfaction

**Fig. 1:**
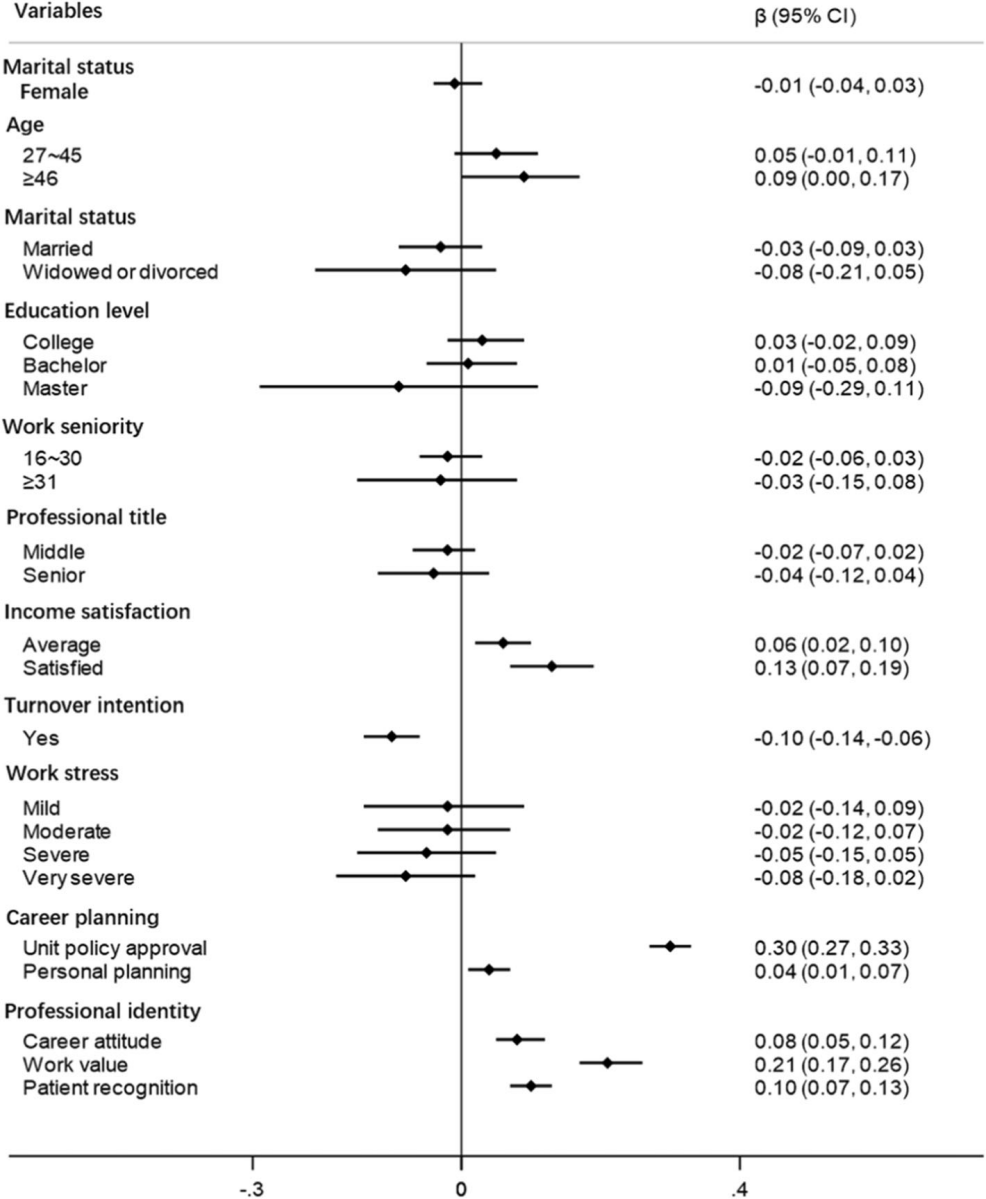
This figure illustrates the β and 95% confidence intervals of independent variables in MLM, and the reference group was male, ≤26, single, below college, ≤15, junior, dissatisfied, no and no from marital status to work stress, respectively

The Socio-demographic characteristics, career planning and career identify were used as independent variables and the job satisfaction was used as dependent variables in MLM and QR model, respectively. MLM analysis showed that the doctors whose age group ≥46 years had a higher job satisfaction score than that≤26 years (β = 0.09, 95CI%: 0.00, 0.17). Doctors with average income satisfaction and satisfied income satisfaction had a higher job satisfaction scores than those with dissatisfied income satisfaction (β = 0.06, 95CI%: 0.02, 0.10) (β = 0.13, 95CI%: 0.07, 0.19), while those with turnover intention had low job satisfaction (β = − 0.10, 95CI%: − 0.14, − 0.06). The scores of unit policy approval, personal planning, career attitude, work value and patient recognition were positively correlated with the job satisfaction score (β = 0.30, 95CI%: 0.27, 0.33) (β = 0.04, 95CI%: 0.01, 0.07) (β = 0.08, 95CI%: 0.05, 0.12) (β = 0.21, 95CI%: 0.17, 0.26) (β = 0.10, 95CI%: 0.07, 0.13) (Fig. 1).

The results of QR were not entirely consistent with MLM in different job satisfaction score percentiles. Unit policy approval and work value were positively correlated with job satisfaction in each percentiles. PCPs with work seniority ≥31 years, severe and very severe work stress had negative effects on outcome at higher quantiles of the job satisfaction distribution. PCPs with higher income satisfaction, higher career attitude scores and higher patient recognition scores had a positive association with outcome at higher quantiles of the job satisfaction distribution. PCPs with turnover intention had a negative correlation with outcome at lower quantiles of the job satisfaction. Other details were shown in Table 4.

**Table 4:** QR results for accessing the determinants of job satisfaction

		β (95CI%)								
		P10	P20	P30	P40	P50	P60	P70	P80	P90
Gender	Female	0.11 ^1^ (0.03,0.19)	0.05(0.00,0.10)	0.02(−0.05,0.08)	0.02(− 0.04,0.08)	0.00(− 0.06,0.06)	0.00(− 0.05,0.05)	0.00(− 0.04,0.04)	− 0.03(− 0.08,0.02)	− 0.01(− 0.09,0.06)
Age (year)	27 ~ 45	0.05(− 0.08,0.18)	0.03(− 0.05,0.11)	0.08(− 0.02,0.17)	0.07^1^ (0.00,0.15)	0.04(− 0.03,0.11)	0.05(− 0.00,0.10)	0.07(− 0.00,0.14)	0.07(− 0.02,0.16)	0.03(− 0.05,0.12)
	≥46	0.15(− 0.04,0.33)	0.04(− 0.09,0.166)	0.07(− 0.06,0.19)	0.06(− 0.04,0.15)	0.03(− 0.07,0.13)	0.03(− 0.07,0.12)	0.04(− 0.05,0.13)	0.08(− 0.06,0.23)	0.07(− 0.08,0.23)
Marital status	Married	0.05(− 0.07,0.17)	0.05(− 0.05,0.15)	− 0.03(− 0.13,0.07)	− 0.04(− 0.12,0.03)	− 0.0(− 0.09,0.05)	− 0.01(− 0.06,0.04)	− 0.03(− 0.10,0.03)	−0.00(− 0.09,0.09)	−0.04(− 0.13,0.06)
	Widowed or divorced	0.08(− 0.18,0.35)	− 0.01(− 0.26,0.23)	−0.07(− 0.27,0.14)	−0.13(− 0.28,0.02)	−0.09(− 0.24,0.07)	0.01(− 0.10,0.12)	− 0.04(− 0.15,0.07)	−0.02(− 0.18,0.13)	−0.14(− 0.35,0.07)
Education level	College	−0.01(− 0.14,0.12)	− 0.05(− 0.14,0.04)	−0.04(− 0.12,0.04)	0.00(− 0.06,0.06)	0.02(− 0.05,0.09)	0.04(− 0.03,0.11)	0.08^1^ (0.02,0.14)	0.05(− 0.03,0.14)	− 0.02(− 0.11,0.08)
	Bachelor	0.00(− 0.11,0.11)	− 0.03(− 0.13,0.06)	−0.04(− 0.10,0.03)	−0.00(− 0.06,0.05)	−0.01(− 0.08,0.06)	−0.00(− 0.06,0.06)	0.05(− 0.01,0.10)	0.04(− 0.06,0.13)	− 0.03(− 0.14,0.08)
	Master	− 0.20(− 0.62,0.23)	−0.13(− 0.52,0.26)	−0.10(− 0.39,0.20)	− 0.09(− 0.37,0.20)	−0.15(− 0.41,0.10)	−0.05(− 0.26,0.17)	−0.05(− 0.25,0.15)	−0.09(− 0.44,0.26)	− 0.14(− 0.53,0.26)
Work seniority (year)	16 ~ 30	0.01(−0.08,0.11)	− 0.04(− 0.10,0.03)	−0.03(− 0.10,0.05)	−0.02(− 0.09,0.05)	− 0.04(− 0.09,0.02)	−0.04(− 0.11,0.02)	−0.02(− 0.08,0.04)	−0.05(− 0.11,0.01)	−0.01(− 0.10,0.09)
	≥31	0.12(−0.02,0.25)	0.03(− 0.11,0.17)	− 0.01(− 0.14,0.12)	0.04(− 0.14,0.21)	0.02(− 0.09,0.14)	− 0.02(− 0.13,0.08)	−0.03(− 0.13,0.08)	− 0.08(− 0.20,0.05)	− 0.21^1^ (− 0.35,-0.07)
Professional title	Middle	−0.05(− 0.15,0.05)	−0.01(− 0.08,0.05)	−0.05(− 0.10,0.01)	−0.03(− 0.09,0.03)	−0.01(− 0.06,0.05)	0.01(− 0.05,0.07)	0.01(− 0.04,0.07)	0.01(− 0.06,0.08)	0.00(− 0.12,0.12)
	Senior	− 0.15(− 0.39,0.09)	−0.06(− 0.19,0.08)	−0.08(− 0.18,0.03)	−0.03(− 0.14,0.07)	0.01(− 0.10,0.11)	0.02(− 0.09,0.13)	0.05(− 0.06,0.16)	0.03(− 0.09,0.14)	0.03(−0.13,0.18)
Income satisfaction	Average	0.13^1^ (0.05,0.21)	0.07^1^ (0.00,0.15)	0.07^1^ (0.01,0.12)	0.06^1^ (0.02,0.11)	0.03(−0.01,0.07)	0.03(−0.01,0.06)	0.03(− 0.02,0.07)	0.06^1^ (0.01,0.12)	0.02(− 0.04,0.08)
	Satisfied	−0.01(− 0.26,0.24)	0.08(− 0.05,0.21)	0.11(− 0.01,0.23)	0.14(0.01,0.27)	0.17^1^ (0.08,0.26)	0.15^1^ (0.07,0.23)	0.12^1^ (0.04,0.120)	0.18^1^ (0.10,0.27)	0.18^1^ (0.07,0.29)
Turnover intention	Yes	−0.14^1^ (− 0.24,-0.03)	−0.12^1^ (− 0.20,-0.04)	−0.14^1^ (− 0.20,-0.08)	−0.11^1^ (− 0.17,-0.06)	−0.09^1^ (− 0.14,-0.03)	−0.06^1^ (− 0.11,-0.01)	−0.07^1^ (− 0.11,-0.03)	−0.05(− 0.11,0.00)	−0.07(− 0.15,0.02)
Work stress	Mild	0.02(−0.31,0.35)	− 0.05(− 0.45,0.35)	−0.04(− 0.19,0.10)	−0.00(− 0.14,0.13)	−0.11(− 0.27,0.06)	−0.09(− 0.25,0.06)	−0.13(− 0.29,0.04)	−0.11(− 0.25,0.03)	−0.05(− 0.21,0.11)
	Moderate	0.11(−0.15,0.37)	− 0.02(− 0.37,0.33)	−0.03(− 0.15,0.08)	0.02(− 0.12,0.17)	−0.07(− 0.25,0.11)	−0.09(− 0.27,0.08)	−0.14(− 0.29,0.01)	−0.11(− 0.26,0.03)	−0.04(− 0.22,0.15)
	Severe	0.12(−0.15,0.39)	− 0.04(− 0.39,0.31)	−0.05(− 0.17,0.07)	0.01(− 0.11,0.13)	−0.09(− 0.24,0.07)	−0.11(− 0.25,0.04)	−0.16^1^ (− 0.30,-0.02)	−0.16^1^ (− 0.28,-0.03)	−0.09(− 0.25,0.07)
	Very severe	0.15(−0.16,0.46)	− 0.05(− 0.41,0.32)	−0.08(− 0.20,0.04)	−0.03(− 0.17,0.10)	−0.14(− 0.31,0.03)	−0.16(− 0.33,0.01)	−0.18^1^ (− 0.35,-0.02)	−0.16^1^ (− 0.31,-0.01)	−0.08(− 0.27,0.11)
Career planning	Unit policy approval	0.30^1^ (0.23,0.37)	0.32^1^ (0.28,0.37)	0.33^1^ (0.29,0.37)	0.32^1^ (0.29,0.35)	0.32^1^ (0.28,0.35)	0.31^1^ (0.27,0.36)	0.30^1^ (0.26,0.35)	0.27^1^ (0.22,0.31)	0.26^1^ (0.20,0.31)
	Personal planning	0.02(−0.04,0.08)	− 0.01(− 0.05,0.04)	0.01(− 0.03,0.05)	0.01(− 0.02,0.05)	0.04(− 0.01,0.08)	0.04(−0.00,0.08)	0.04(0.00,0.08)	0.03(−0.00,0.07)	0.02(− 0.02,0.05)
Professional identity	Career attitude	0.01(−0.05,0.07)	0.06(−0.00,0.12)	0.05^1^ (0.00,0.10)	0.06^1^ (0.02,0.11)	0.09^1^ (0.03,0.14)	0.10^1^ (0.06,0.14)	0.10^1^ (0.06,0.15)	0.13^1^ (0.07,0.19)	0.14^1^ (0.07,0.21)
	Work value	0.30^1^ (0.23,0.36)	0.23^1^ (0.17,0.30)	0.22^1^ (0.17,0.272	0.23^1^ (0.17,0.30)	0.23^1^ (0.17,0.30)	0.23^1^ (0.15,0.30)	0.24^1^ (0.18,0.31)	0.22^1^ (0.15,0.29)	0.25^1^ (0.19,0.31)
	Patient recognition	0.03(−0.03,0.08)	0.07^1^ (0.02,0.11)	0.10^1^ (0.06,0.14)	0.11^1^ (0.07,0.15)	0.12^1^ (0.07,0.16)	0.14^1^ (0.10,0.19)	0.16^1^ (0.10,0.21)	0.16^1^ (0.09,0.23)	0.16^1^ (0.08,0.23)

Note: ^1^ *P* < 0.05; The reference group was male, ≤26, single, below college, ≤15, junior, dissatisfied, no and no from marital status to work stress, respectively

The satisfaction scores per decile group were 2.54, 2.77, 3, 3.08, 3.15, 3.38, 3.54, 3.85 and 4.08, respectively

## Discussion

In our result, we found that the job satisfaction of PCPs was not high after the new health care reform, which was consistent with many domestic studies [16–18]. Despite numerous efforts, the progress in improving doctors’ satisfaction had been little. Our study found that doctors who were with an age ≤ 26-year-old, with higher income satisfaction, with lower turnover intention, with higher unit policy approval, with better personal planning, with higher career attitude, with higher work value, and with higher patient recognition would have higher job satisfaction. Efforts should be made in these aspects.

In this study, unit policy approval has the greatest impact on PCPs’ job satisfaction. Consistent with other studies, we note that in the career planning dimensions, PCPs who have higher unit policy approval scores and higher personal planning scores have higher job satisfaction scores [19]. Despite the financial benefits, career planning is considered as a psychological contract between the employee and the employer [20]. And it is acted as an activity of human resource management practices aimed at improving employees’ personal growth and performance [21]. If the health care authorities couldn’t invest their doctors in planning and developing their career at the right speed or intensity, doctors may behave dissatisfaction, low productivity and ultimately changing jobs. On the contrary, doctors who are satisfied with their career planning and development shall display highly committed towards their health care authorities and the departments shall in return, be able to ensure long term retention of skilled and loyal doctors. A survey conducted in Indian public and private health sectors investigating doctors’ job satisfaction noted that although health professionals working in public hospitals had reasonable working hours, relatively higher wages and other benefits, private hospital health professionals had higher job satisfaction due to positive unit policy approval and personal planning [22]. Therefore, we suggest more non-financial incentives such as additional training opportunities for professional growth and promotion be created among doctors to improve their career planning. Further studies are needed to ascertain the degree to which career planning is associated to job satisfaction.

In the professional identity dimensions, our study reveals that doctors who have higher career attitude scores, higher work value scores, and higher patient recognition scores would have higher job satisfaction. A great deal of scientific researches have predicted a positive relationship between professional identity and job satisfaction [23, 24]. This can be explained by the congruence that professional identity fosters a belief that an individual’s career will be rewarded by the profession, which in turn generate a higher level of job satisfaction. Previous study noted that meeting patient expectations was highly correlated with physician satisfaction at primary level [10]. This may be because there is a positive correlation between patient recognition and feeling of pride or personal accomplishment. In Chinese traditional culture, medical staff belong to a noble profession. When an individual has obtained sufficient professional identity, even in an unfavorable work environment, the individual will maintain a high degree of work engagement and obtain better job satisfaction [25]. One study specific to convention and exhibition industry professionals in Asia reported that job satisfaction between respondents with strong/weak professional identity were not significant. This can be explained by the fact that job category may partially influence the results. In addition, as the “gatekeeper” of residents’ health, PCPs is engaged in preventive care and overall health management, so doctor-patient relationship largely determines job satisfaction [26].

Mirroring the results in similar studies, the present study indicated that there was negative correlation between job satisfaction and turnover intention [27, 28]. People do not trust the professional capabilities of PCPs. Not to mention surgery, and dealing with critically ill patients, even some medicines at the primary care level cannot be equipped [29]. People choose to see a doctor in tertiary hospitals rather than in a local one. PCPs felt it difficult to meet patients’ demand and the sense of professional accomplishment of PCPs is getting weaker and weaker, then they intended to leave their job, thus creating a vicious cycle.

It is not surprising that in our study, PCPs with higher income satisfaction are more likely to have higher job satisfaction. This can be explained by the fact that higher income satisfied their living needs and relieved their worries about the risks later. Income is considered essential to providing health services [30]. These low-income PCPs are unable to meet the living expenses and have a sense of dissatisfaction with the current work. They may hope to pursue a high income by finding a job in a city hospital or changing careers. Similar positive association has been reported between income satisfaction and job satisfaction [31–33]. In China, primary health care institutions have limited autonomy and flexibility, which has led them to be greatly affected by basic wages. This has harmed doctors’ job satisfaction to a certain extent. From a global perspective, regions with high satisfaction levels of primary health care doctors are usually areas with high economic development [10]. A large part of this is due to their strong financial security capabilities in providing people-oriented, public welfare services. The government should streamline administration and institute decentralization and raise PCPs’ income. Meanwhile, promoting the implementation of performance-based pay in primary care institutions which fully reflects the value of talents may improve PCPs’ job satisfaction.

The present finding reveals that PCPs with an age older than 46 years result in a higher job satisfaction compared with doctors who are less than 26 years old. The results are consistent with some previous studies, which have proved that the older the age was, the higher satisfaction degree was got [34, 35]. It could be explained by the fact that doctors with an older age had worked for a long time at their own practice, so they probably had paid off the house loan or other debt. However, the younger had to face the economic stress. Furthermore, older doctors were used to their current working environment, thus leading to higher job satisfaction than the young. Prior study demonstrated that with the increase of working age, PCPs had gained more experience in professional skills, handling doctor-patient relationship and communication, working environment and patient outcomes, so they were more possibly to have a higher professional identity, thus leading to higher job satisfaction [36].

The results of QR were not completely consistent with MLM. The results of MLM analysis was relatively simple as only the overall impact of all possible influencing factors on job satisfaction was analyzed which was greatly affected by the linear regression assumptions. If the conditions was not met, the results might differ from the actual situation. QR can fully describe the overall picture of the conditional distribution of the interpreted variable, rather than just analyzing the conditional expectation of the interpreted variable, and can also analyze how the explanatory variable affects the median and quantile of the interpreted variable. Moreover, the quantile regression does not require strong assumptions for the error term, so in terms of abnormal distribution, the quantile regression coefficient estimator is more robust. Taking the influence of turnover intention on job satisfaction as an example, compared with the results of traditional regression methods (β = − 0.1, *p* < 0.05), the QR further compared the degree of influence at different percentile points (β:-0.14 ~ − 0.07, *p* < 0.05). QR showed that the influencing factors affecting PCPs’ satisfaction were different at different quantiles of satisfaction, so targeted measures should be implemented.

### Strengths and limitations

The strength of this investigation is that our sample come from 11 provinces in the western China which covers nearly 70% of the country’s territory. The sample coverage is wide and the population is representative. Apart from the Han people, there are more than fifty minorities living in the western region, which is the most concentrated in China [37]. Meanwhile, to our knowledge, the research on job satisfaction for PCPs is predominantly conducted in developed countries and less in developing countries. Therefore, our research results can provide policy advice for poverty alleviation and supporting PCPs in western areas, and further satisfy the equity principle highlighted in the new medical reform. Especially, our study used QR which could avoid the traditional linear regression based on ordinary least squares underestimating or overestimating the effects of independent variables.

Several limitations of our study must be considered. First, since this survey was targeted at western PCPs and the selection bias couldn’t be avoided, this result are hard to extrapolated to national level or other countries. Second, the survey focused on limited factors that might impact, we didn’t assess all the possible key determinants like group cohesion [38], and institutional context [39], so further studies are needed to examine more factors. Third, as the measurement of satisfaction were obtained by self-reported questionaires, the recall bias could not be eliminated. Fourth, it is still impossible to summarize the influencing factors of job satisfaction in the western china based on the results of one survey. Fifth, we don’t get a corroborated causal link through this research, since this is a cross-sectional study, the significant results could be chance results. Yet, the present study provides us the potential causes and we need to further confirm the links. Sixth, we conducted the study in rural settings, which had different characteristics from urban settings. In this regard, urban information should be added to compare the difference between them. Meanwhile, we don’t compare the difference between respondents and non-respondents as we did not collect the related information. Seventh, the data used in this study is old. However, considering the satisfaction of PCPs in western China has not changed much in recent years and the sample size, the results of this study still had a strong reference value. Another disadvantage is that this study used a face-to-face questionnaire survey. In this way, the results of the survey may be affected by social desirability [40], leading to biased results. Nevertheless, the current study provides the updated information and the potential determinants of job satisfaction among PCPs in this geographical region.

## Conclusion

This study showed that job satisfaction of PCPs in western China is not high with an overall satisfaction score was 3.26 ± 0.68 on a one to five scale. Meanwhile, it provides relevant evidence from rural areas in western China and adds to the growing international literature on the satisfaction of PCPs. The MLM and QR discussed are not entirely consistent, the latter one provided more information and robust results. The study of QR applied to the job satisfaction among PCPs has a high application value, and it is recommended to be widely applied in scientific researches. Therefore, financial and non-financial incentives should be considered to implement for healthcare managers to increases satisfaction among PCPs. Further studies may look further into unexplored factors which are possible contribute to the PCPs’ job satisfaction. In order to reduce social desirability bias, some methodology like anonymity, grouped answer, and question randomization can be used in the future investigations. Longitudinal investigations are needed for further conforming the causality. Moreover, we could conduct multi-centre studies in rural PCPs of different provinces and the findings can be extrapolated at the national level.

## Data Availability

The data used and/or analyzed during the study are available from the corresponding author on reasonable request.

## Abbreviations

PCPs: Primary care physicians
MLM: A multilevel model
QR: Quantile regression
